# Seasonal coat-colour moulting phenology of snowshoe hares in a Yukon boreal forest undergoing climate change

**DOI:** 10.1098/rsos.250662

**Published:** 2025-11-12

**Authors:** Yadav P. Ghimirey, Alice J. Kenney, Charles J. Krebs, Madan K. Oli

**Affiliations:** ^1^ Department of Wildlife Ecology and Conservation, University of Florida, Gainesville, FL, USA; ^2^ Department of Zoology, University of British Columbia, Vancouver, British Columbia, Canada; ^3^ 211 Old Highway Road, Kluane Lake, YT Y0B 1H0, Canada

**Keywords:** boreal forest, climate change, coat-colour mismatch, moulting, *Lepus americanus*, local climate dynamics

## Abstract

Climate change is slowly influencing boreal forest ecosystems, with rising temperatures and altered snow conditions driving phenological shifts in many plant and animal species. Using 7 years (2016–2022) of camera trap data from the Kluane Lake region, Yukon, we quantified seasonal moulting phenology and coat-colour mismatch in snowshoe hares. Autumn moult started between 28 September and 3 October and completed between 5 and 11 November, with the mean moult duration ranging from 36 to 43 days. Spring moult initiated between 12 April and 27 April and completed between 16 May and 27 May, with moult duration ranging from 24 to 38 days. Contrary to our expectations, there was no evidence of delayed or advanced moulting phenology over this 7-year period. The mismatch between snowshoe hare coat colour and background showed an increasing trend and average whiteness of the snowshoe hare coat in autumn declined. Temperature and snow variables influenced various aspects of seasonal moulting phenology, in some cases in the opposite direction. Long-term studies utilizing intrinsic and high-resolution microclimatic data and behavioural observations are needed to understand how moulting phenology and mismatch affect predator–prey dynamics and snowshoe hare demography and population dynamics as climate change continues.

## 1. Introduction

The Intergovernmental Panel on Climate Change synthesis report of 2023 asserts with high confidence that Earth’s surface temperatures have risen more rapidly over the past two decades than at any point in the last 2000 years [1]. This accelerated warming has impacted ecosystems globally, with circumpolar regions experiencing the most pronounced effects [2–4]. These impacts extend across all levels of ecological organization [5], significantly influencing species distributions, abundance, population dynamics and interspecific interactions [6–9]. Phenological shifts have also been observed in numerous species of plants and animals in response to changing climate [6–10]. Whereas phenological changes can be adaptive in some cases and buffer against some adverse effects of changing climate, they can also create phenological mismatches potentially reducing species fitness [11,12].

The snowshoe hare *Lepus americanus*, a species well known for 10-year population cycles [13,14], is a keystone herbivore in many North American boreal forests, both in terms of herbivore biomass and their importance in the food web [15,16]. A wide range of mammalian and avian predators in these boreal forests rely on snowshoe hares as a primary food source, with their reproductive success closely linked to snowshoe hare abundance [15,16]. Snowshoe hares change coat colour seasonally, shifting from brown to white during the autumn moult and back to brown during the spring moult. The white winter coat in snowshoe hares is thought to be an adaptation to minimize predation risk via crypsis [17,18]. There is good evidence that snowshoe hares experience higher mortality when their coat colour is mismatched with the background environment [19–21].

Temperature, precipitation and snow duration and depth in northern circumpolar regions, the primary distributional range of snowshoe hares, are changing in response to warming climate; these changes can affect snowshoe hare moulting phenology [17,22,23]. Increased temperatures (thus, shorter snow duration) are thought to advance the dates of initiation and completion of moulting in spring, and vice versa in autumn [24], and potentially increase the coat-colour-background mismatch [23,25]. The increased mismatch can increase predator attacks on hares [26] and reduce winter survival [19–21], potentially leading to the dampening or collapse of snowshoe hare population cycles. Collapse of snowshoe hare population cycles will have a devastating and cascading effect across the boreal forest ecosystems due to their importance in the boreal forest food chain [27,28].

Using 7 years (2016–2022) of continuously operating camera trapping data, we quantified the exact timing of seasonal moulting phenology and attempted to discern factors influencing initiation and completion of seasonal moulting in snowshoe hares in the Kluane Lake region, Yukon, Canada. Recent studies suggest altered moulting phenology and an increased coat-colour-background mismatch in seasonally moulting lagomorphs [26,29,30]. Based on the observed changes in climatic conditions (temperature, precipitation and snow characteristics) and findings of previous studies [21,29], in the southern part of the snowshoe hare range, we hypothesized that: (i) rising temperatures and decreasing snow duration and depth influence the timing and duration of the spring and fall moults in Kluane. Furthermore, there is evidence of limited plasticity in lagomorph moulting phenology [30,31] and changes in snow duration and depth [32] can potentially exacerbate the coat-colour-background mismatch. Thus, we also hypothesized that: (ii) the temperature and snow conditions will influence coat-colour mismatch with the background. Thus, we tested the following predictions: (i) the increase in temperature (minimum, maximum and mean daily temperature) would result in spring moult occurring earlier and autumn moult later; and (ii) rising temperature and decreasing snow-related variables (snow cover, snow duration and snow depth) increase the coat-colour background mismatch.

## 2. Methods

### 2.1. Study area

**Figure 1.**
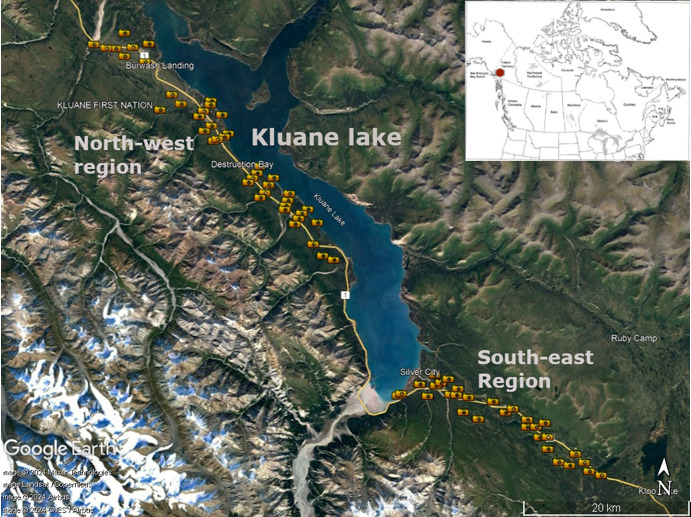
Locations of 72 camera trap stations (indicated by camera icons) that contributed to the snowshoe hare moulting phenology data. The camera traps are close to Kluane lake, known also as Lhù'ààn Mân, located in the south-western corner of Yukon territory, Canada (61°N, 138.5°W) indicated by the red dot in the map inset. Camera traps were set up in two distinct areas of the lake surroundings, the north-west and the south-east. The figure is adapted from [33].

This study was conducted in the boreal forest near Kluane Lake (Lhù’ààn Mân), southwestern Yukon, Canada (figure 1). The climate is dry and cold with~30 cm of annual rainfall, 30%–40% of which falls as snow [16]. Summers are warm with long days extending up to 19 h. Average temperature varies widely, ranging from 11°C in June to −21°C in January [16]. Vegetation is dominated by white spruce *Picea glauca*, with trembling aspen *Populus tremuloides* and balsam poplar *Populus balsamifera* as secondary species. The understory consists of willows *Salix spp*., dwarf birch *Betula grandulosa*, soapberry *Shepherdia canadensis*, bearberry *Arctostaphylos uva-ursi*, crowberry *Empetrum nigrum*, lingonberry *Vaccinium vitis-idaea*, toadflax *Geocaulon lividum* and red bearberry *Arctostaphylos rubra* [15,16]. The Kluane Lake region supports ~150 bird and ~50 mammal species (https://parks.canada.ca/pn-np/yt/kluane/nature/mammiferes-mammals).

### 2.2. Camera trap surveys

During 2016–2022, we conducted a camera-trapping study at two locations in southwestern Yukon: northwest of Kluane Lake (where 42 cameras were deployed) and southeast of Kluane Lake (where 30 cameras were deployed) (fig. 1 from [33]). In 2016 and 2017, we used Reconyx PC900 (Reconyx Inc., Wisconsin, USA), Bushnell Trophy Cam (Bushnell Outdoor Products, Kansas, USA) and Scoutguard SG570 (HCO Outdoor Products, Georgia, USA) cameras; Bushnell and Scoutguard cameras were replaced by Reconyx HP2X cameras in 2018. All cameras were motion-sensitive and were mounted on tree trunks 24–70 cm above ground in secured locked boxes, separated from one another by ≥ 1 km. Cameras were randomly placed with respect to the vegetation types on game trails or openings that provided a field of view between 3 and 49 m^2^. All cameras were equipped with infrared flash to minimize disturbance to the target species. No baiting was used. Batteries were replaced once a year; the location of camera and its settings remained unchanged during the study. Cameras were programmed to take photographs continuously supplemented by one time lapse photograph at noon every day. Photographs were tagged manually to record relevant information, and the photographic database was managed using a Microsoft Access database [33].

### 2.3. Quantification of coat colour and coat-colour-environment mismatch

In Kluane, snowshoe hares are entirely brown until the end of August and pure white until the end of February; they are in various moult stages during September–November and March–May. For the analysis of spring moult, we selected camera trap images taken between 1 March and 31 May; for the analysis of autumn moult, we used images taken between 1 September and 30 November. It is possible that an individual hare can be photo-captured in the same camera trap multiple times in the same moulting stage. In such cases, identifying individual snowshoe hares from photographs is difficult, raising concern of samples not being independent. Hence, we considered only one image per camera trap per day (the first image) as an independent event. For each independent image, we recorded camera ID, location, date and time. We visually estimated the percent white coat at 10% increments (using a standardized scoring protocol developed by C. J. Krebs, unpubl.), and the percent snow cover on the ground (background snow cover in images with snowshoe hares) at 5% increments. Two hares in a single image were considered two independent events and were scored independently. We calculated the percent coat-colour-background mismatch as the difference between the percent white coat of snowshoe hare and percent white of the snow cover as estimated from the same camera images.

### 2.4. Analysis of percent white, initiation and completion of moulting

We converted percent white to proportion that ranged between 0 and 1. We then fitted a generalized linear mixed model (GLMM) using proportion white as the response variable with beta family of distribution and logit link function [34]. In all analyses, we included the camera trap as random effect to account for the possible lack of independence of images taken by the same cameras.

We aimed to quantify the seasonal moulting progression of snowshoe hare using information on the coat whiteness, and to test for annual variation in the timing of moulting. Hare coat whiteness peaking later in the autumn would indicate that hares are moulting later in the season and vice versa. For this, we fitted GLMM (function *glmmTMB* in package *glmmTMB* with beta family) [35] with ordinal day (the number of days since the start of a year) and year as predictors and proportion white as a response variable to quantify seasonal moulting progression and to test for annual variation in moulting progression. We then predicted the mean and 95% CIs of the whiteness based on the best performing model using the function ‘*ggpredict’* from the r package ‘*ggeffects*’ [36] and used these predictions to construct the moulting progression curves for each season and year.

In seasonally moulting species, moult phenology is principally entrained by photoperiod, with temperature and snow conditions modulating the rate and progression of moult [17,23,37–39]. Thus, we obtained temperature (daily minimum, daily maximum and daily mean) and snowfall data (daily snow depth and snow duration) from Haines Junction Climate Station, Yukon (https://weather.gc.ca/canada_e.html) and GIS data (elevation and aspect rasters) from the Government of Canada web portal (https://open.canada.ca/data). We considered the percent snow cover score from images as an index of ground snow cover. All continuous covariates were standardized to mean zero and standard deviation of one. We also calculated pairwise Pearson’s correlation coefficients; we did not include highly correlated covariates (*r* > 0.7) in the same model to avoid problems associated with multicollinearity [40]. A detailed description of covariates used in the analysis is provided in the electronic supplementary material, S1 and table S1.

To test for the climatic influences on the whiteness in snowshoe hare coat colour, we used GLMMs with a beta family and logit link, implemented through the R package '*glmmTMB*' and function ‘*glmmTMB*’ [35,41]. We initially ran univariate models for each season separately, testing for the singular effect of each covariate. We then tested the additive effects of covariates identified to be influential in the univariate analyses. Models were built to test our hypotheses regarding factors influencing autumn and spring moulting patterns.

We used a similar modelling strategy to analyse the influence of environmental covariates on the timing of initiation and completion of moulting in each season. First, we classified moult stages using the proportion of white coat. For autumn, we assumed that moult began when the coat reached 0.1–0.2 white and was complete at 0.8–0.9 white; values between 0.2 and 0.8 were considered moult in progress. For spring, we applied the reverse: we assumed that moult had begun when the coat was 0.8–0.9 white, and that moult was complete when the coat was 0.1–0.2 white; moult was assumed to be in progress at the intervening values of coat-colour whiteness [21,29]. Unless stated otherwise, thresholds are inclusive of the stated bounds (e.g. 10% and 20%). We defined independent moulting initiation and completion events if images captured at different camera traps on the same day met the predefined thresholds of mean coat whiteness, or if images captured at the same camera trap were separated by ≥3 days. These criteria assumed that it would take at least 3 days for the coat colour to change by ~10%, and an individual would not be detected with the same mean coat whiteness three days apart in the same camera trap. For each independent initiation and completion event, we recorded the Ordinal date, from which we calculated the mean ordinal day of both initiation and completion for each season and year, and their 95% CIs.

To understand how initiation and completion of moulting were influenced by the climatic covariates, we used GLMMs, implemented through the R package *‘glmmTMB*‘ and function ‘*glmmTMB*’ with Gaussian family of distribution [35,41]. We first ran univariate models for each season separately, testing for the singular effect of each covariate. Using covariates identified as influential in the univariate models, we then tested for their additive effects to assess their effects on initiation and completion of moulting for autumn and spring seasons separately.

### 2.5. Analysis of coat-colour-background mismatch

We considered mismatch as occurring if the difference between the percent white in the coat and the percent snow cover exceeded 10%; this threshold was chosen because the underside of snowshoe hare legs and belly often remain completely or partially white throughout the year [22]. We quantified mismatch in two different ways; first, we grouped the observed mismatch into three broad categories: low (10%–30% mismatch), medium (30%–60% mismatch) and high (>60% mismatch) [42,43]. Temporal variation in mismatch was assessed based on the proportion of mismatch categories in each year of the study. Second, we ran GLMM with proportion mismatch as response and the interaction of ordinal day and year. We used the fitted model to plot the mismatch progression and quantify mismatch as a function of ordinal day.

To test covariate effects on mismatch, we modelled proportion mismatch as a function of the singular and additive effects of climatic variables in the model. As above, we used GLMMs with beta family and logit link function [34]. Analysis and modelling of mismatch and test of covariate effects proceeded as described above, except that we also included the quadratic effects of covariates to account for the potentially nonlinear relationship between the covariates and mismatch as the seasonal moulting progressed.

We employed an information-theoretic approach for model comparison and statistical inference using Akaike information criterion corrected for small sample size $\left(AIC_{c}\right)$ as a measure of model parsimony, and $AIC_{c}$ weight as a measure of relative support in all analyses [44]. Covariate effects were assessed by comparing $AIC_{c}$ for models with and without covariate(s), and by checking to see if 95% CIs for regression coefficient(s) overlapped zero. All analyses were performed in the R (4.4.1) computing environment [41].

## 3. Results

Between 2016 and 2022, we recorded 6,126 independent observations of snowshoe hares, with the annual number of observations ranging from 57 in 2022 (low phase of the snowshoe hare population cycle) to 1965 in 2017 (peak phase of the population cycle; electronic supplementary material, S1, table S2). Observations in autumn (*n* = 3941) were nearly twice as frequent as those in spring (*n* = 2185). The highest and lowest number of monthly observations were made in September (*n* = 1475) and March (*n* = 521), respectively.

### 3.1. Seasonal coat-colour moulting

**Figure 2.**
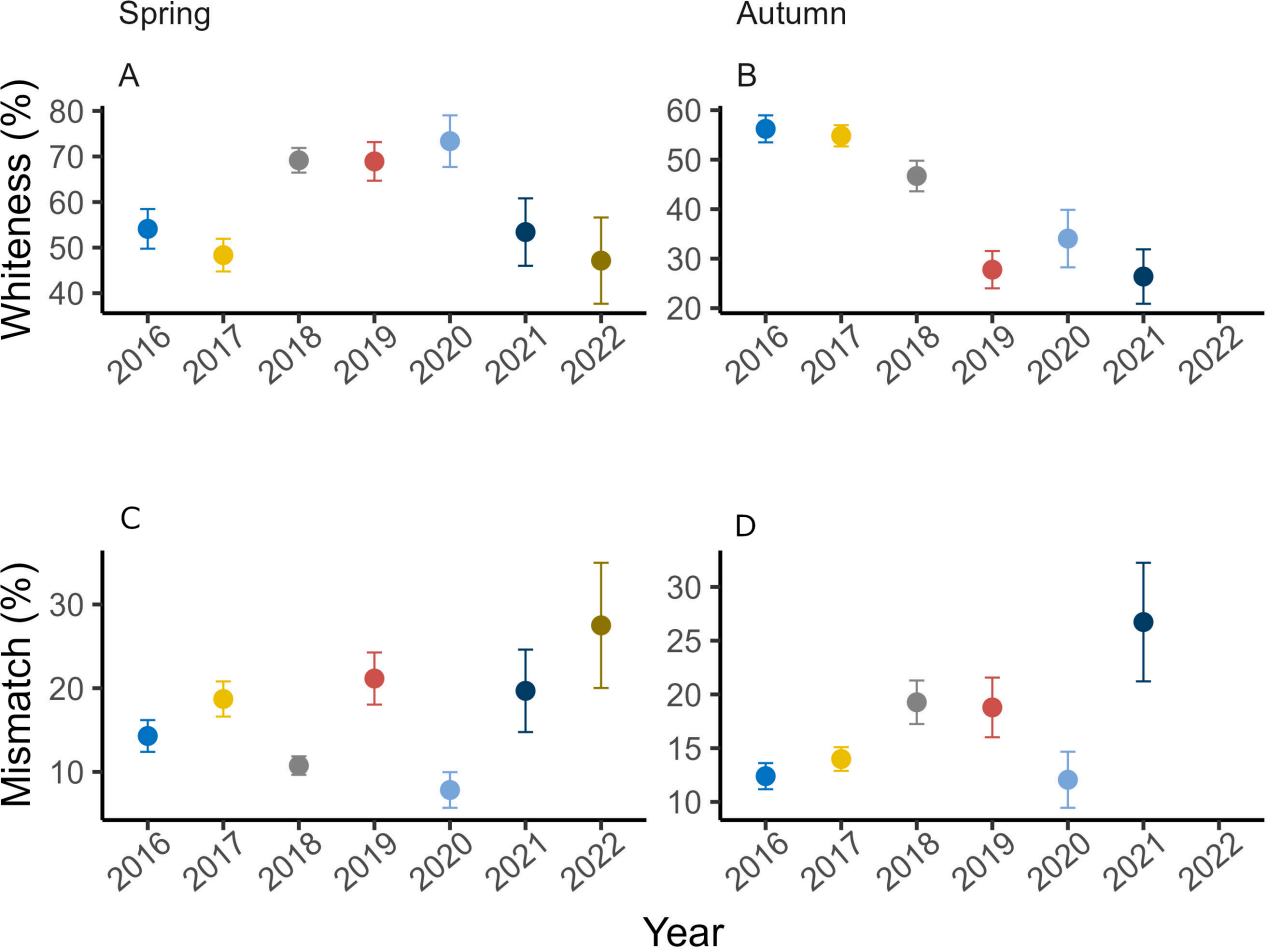
Mean percent whiteness (A: Spring and B: Autumn) and mean percent mismatch (C: Spring and D: Autumn) in snowshoe hare coat during the study period (2016–2022) in Kluane lake region in northern Canada. The upper and lower ends of error bars represent the 95% CI of the mean.

**Figure 3.**
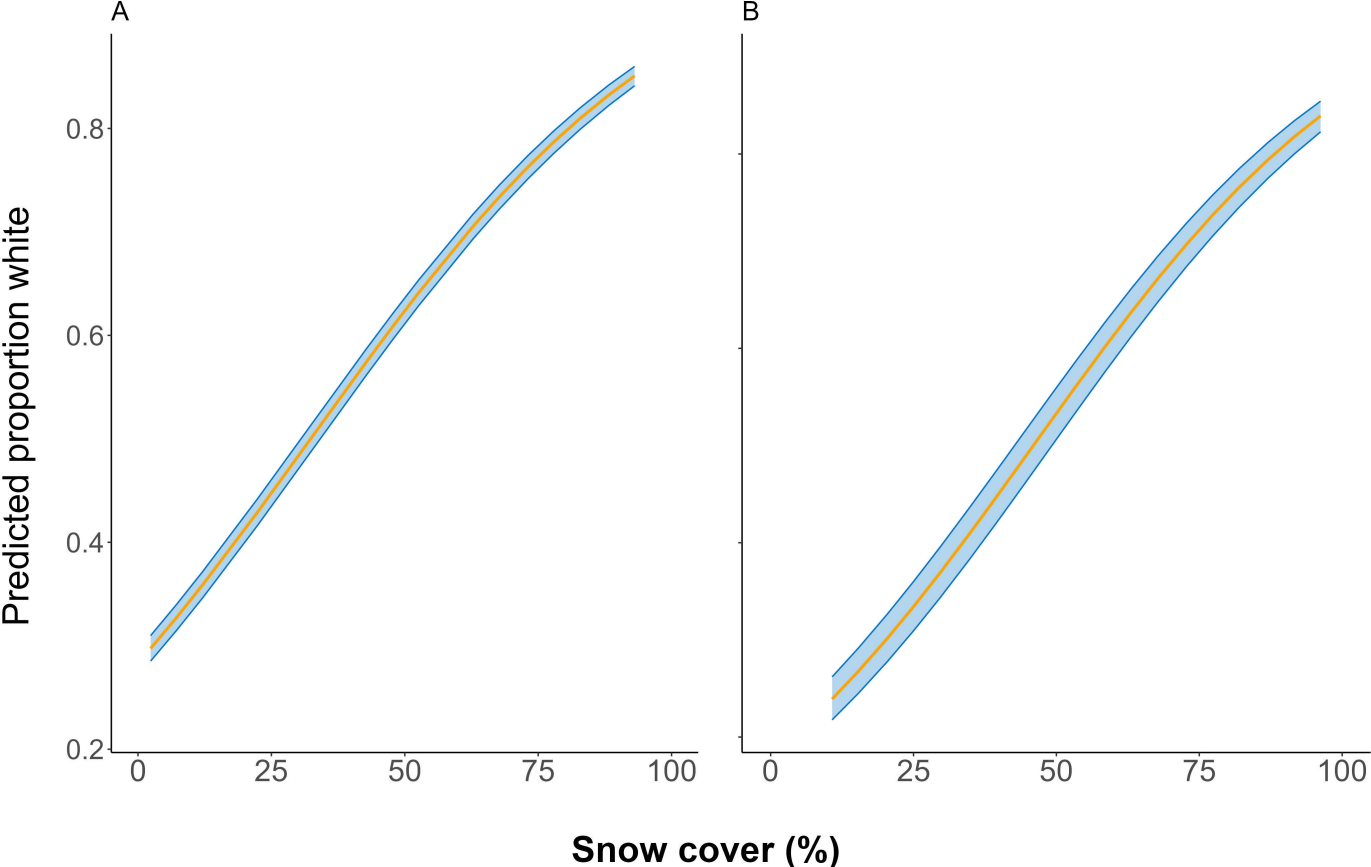
The influence of covariates on snowshoe hare coat whiteness in the Kluane Lake region, Yukon during (A) spring and (B) autumn season. Blue shaded regions are the 95% CI of the estimates. Y axis provides the predicted proportion white of snowshoe hare’s coat based on the best model on percent white. Only covariates from the top univariate models were selected (electronic supplementary materials, table S3, A1 & B1).

In spring, mean coat whiteness ranged between 47% in 2017 and 73% in 2020, with no discernible temporal trend (figure 2A). When the whiteness was modelled with covariates, we found the model with an additive effect of mean daily temperature, percent snow cover and snow depth to be the most parsimonious (electronic supplementary material S1, table S3). Mean daily temperature negatively influenced coat-colour whiteness, whereas snow cover and depth positively influenced it (table 1A; figure 3A). In autumn, the mean coat whiteness ranged from 58% in 2016 to 36% in 2021 and exhibited a generally declining trend (figure 2B). When modelling the covariate influence, we found maximum daily temperature and snow duration negatively affected coat-colour whiteness, whereas the effect of percent snow cover was positive (table 1A; figure 3B).

**Table 1.** The estimates of the fixed effect coefficients of the top performing models to predict whiteness (electronic supplementary material, S1, table S8 A1 & B1), moulting initiation (electronic supplementary material, S1, table S8 C1 & D1), completion (electronic supplementary material, S1, table S8 E1 & F1) and coat-colour mismatch (electronic supplementary material, S1, table S8, G1 & H1) in Kluane. Covariates included snow duration (S_Dur), snow depth (S_Dep), snow cover (S_Cov), maximum daily temperature (Max_T), mean daily temperature (Mean_T) and minimum daily temperature (Min_T). Estimates are presented in terms of the mean, s.e., lower level of 95% confidence interval (LCL) and upper level of 95% confidence interval (UCL).

event	season	covariate	mean	s.e.	LCL	UCL
whiteness	A) spring	int	0.63	0.02	0.58	0.68
		S_Dep	0.22	0.02	0.17	0.27
		Mean_T	−0.55	0.02	−0.60	−0.50
		S_Cov	1.26	0.02	1.21	1.31
	B) autumn	int	0.17	0.03	0.22	0.32
		S_Cov	0.84	0.03	0.78	0.89
		S_Dur	−0.19	0.02	−0.23	−0.14
		Max_T	−1.11	0.04	−1.19	−1.03
initiation	C) spring	int	100.79	1.39	97.69	103.13
		S_Dep	−10.64	1.90	−14.37	−6.90
		S_Dur	3.13	0.81	1.54	4.72
		Mean_T	14.38	2.73	9.04	19.73
	D) autumn	int	279.44	0.97	277.55	281.33
		S_Cov	2.19	0.90	0.43	3.96
		Mean_T	−20.12	1.25	−22.57	−17.66
completion	E) spring	int	132.03	2.53	127.07	136.99
		S_Dur	1.37	0.83	−0.25	2.98
		Mean_T	12.62	3.49	5.77	19.48
	F) autumn	int	298.65	1.36	295.98	301.33
		S_Dur	2.98	1.22	0.60	5.36
		Max_T	−11.27	1.71	−14.63	−7.91
mismatch	G) spring	int	−1.18	0.09	−1.35	−1.00
		S_Cov	−0.23	0.04	−0.29	−0.16
		I(S_Cov^2)	−0.54	0.07	−0.69	−0.40
		S_Dep	−0.64	0.06	−0.76	−0.53
		I(S_Dep^2)	0.17	0.03	0.12	0.22
		Mean_T	−0.27	0.07	−0.41	−0.13
		I(Mean_T^2)	−0.32	0.06	−0.44	−0.19
	H) autumn	int	−0.49	0.13	−0.75	−0.23
		S_Cov	−0.85	0.04	−0.92	−0.78
		I(S_Cov^2)	−0.82	0.11	−1.04	−0.61
		S_Dep	−0.43	0.12	−0.66	−0.19
		I(S_Dep^2)	−0.19	0.17	−0.54	0.15
		Max_T	−1.21	0.05	−1.31	−1.11
		I(Max_T^2)	−0.87	0.03	−0.94	−0.80

**Figure 4.**
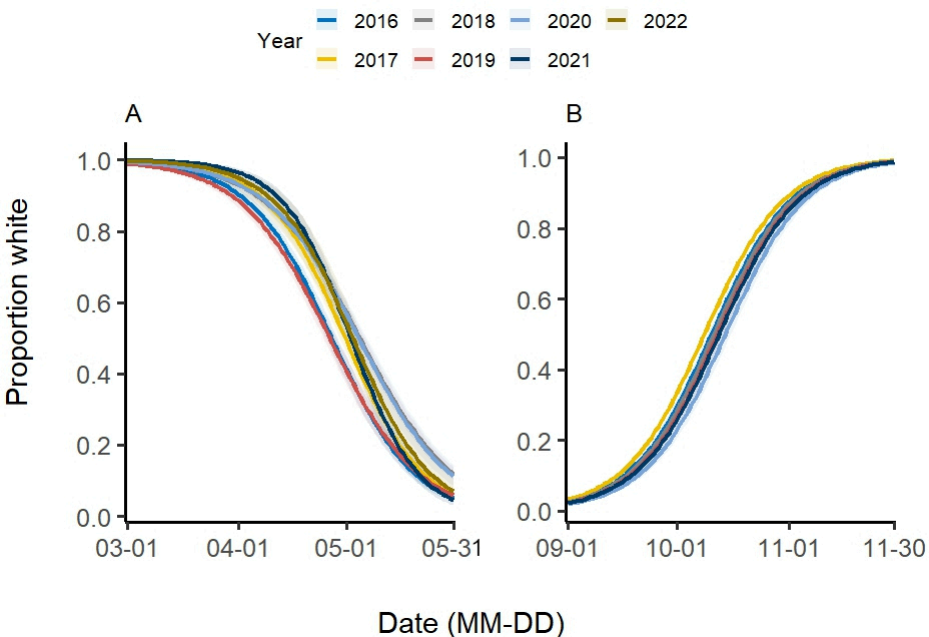
Coat-colour moulting progression of snowshoe hare in (A) spring and (B) autumn season in Kluane lake region, Yukon, Canada from the year 2016 to 2022. The lines of different colours represent the predicted moulting progression for each specific year and shaded region is the 95% CI for each predicted line.

The earliest observed spring moult initiation occurred between 12 April and 27 April and completed between 16 May and 27 May, with moult duration ranging from 24 to 38 days (table 2). The model predicted spring moult initiated (between 31 March and 12 April) and ended earlier (between 23 and 27 May). Moult initiation varied across years, with earlier initiation in 2016 and 2019; however, we found no evidence for advancing initiation trend (figure 4A). When the initiation date was modelled with covariates, the model containing the additive effects of mean daily temperature, snow depth and snow duration was best supported $\left(AIC_{c}wt=0.94\right)$ (electronic supplementary material, S1, table S3 D1). Mean daily temperature and snow duration positively influenced spring moult initiation, whereas snow duration had a negative influence (table 1A). Moult completion in spring also suggested the absence of annual trend. When the simultaneous influence of covariates on completion date was considered, the model that included additive effects of snow duration and mean daily temperature was better supported $\left(AIC_{c}wt=0.66\right)$ (electronic supplementary material, S1, table S3 F1). The model suggested a positive influence of mean daily temperature and snow duration on spring moult completion date (table 1C).

**Table 2.** The mean observed date of initiation (10% white in autumn and 90% white in spring) and completion (90% white in autumn and 10% white in spring) of moulting in snowshoe hares between 2016 and 2022 in Kluane region, Yukon, Canada. Duration indicates the mean number of days between the initiation and completion of moulting. The dates inside the parentheses indicate the lower and upper values of the 95% CI.

year	season	initiation	completion	duration
2016	spring	13 Apr (10 Apr, 16 Apr)	21 May (19 May, 23 May)	38 (33, 43)
	autumn	29 Sep (27 Sep, 30 Sep)	11 Nov (9 Nov, 12 Nov)	43 (40, 56)
2017	spring	26 Apr (23 Apr, 28 Apr)	23 May (22 May, 24 May)	27 (24, 31)
	autumn	28 Sep (27 Sep, 29 Sep)	8 Nov (7 Nov, 9 Nov)	40 (38, 42)
2018	spring	23 Apr (21 Apr, 25 Apr)	24 May (22 May, 25 May)	31 (27, 34)
	autumn	30 Sep (28 Sep, 2 Oct)	11 Nov (10 Nov, 13 Nov)	42 (39, 46)
2019	spring	12 Apr (9 Apr, 15 Apr)	16 May (13 May, 19 May)	34 (28, 40)
	autumn	3 Oct (30 Sep, 6 Oct)	9 Nov (6 Nov, 11 Nov)	37 (31, 42)
2020	spring	24 Apr (22 Apr, 26 Apr)	20 May (16 May, 24 May)	26 (20, 32)
	autumn	30 Sep (25 Sep, 5 Oct)	9 Nov (5 Nov, 14 Nov)	40 (31, 50)
2021	spring	26 Apr (23 Apr, 29 Apr)	20 May (17 May, 24 May)	24 (18, 31)
	autumn	30 Sep (26 Sep, 4 Oct)	5 Nov (3 Nov, 8 Nov)	36 (30, 38)
2022	spring	27 Apr (20 Apr, 4 May)	27 May (23 May, 31 May)	30 (19, 33)

The earliest observed autumn moult initiated between 28 September and 3 October and completed between 5 November and 11 November; moult duration ranged from 36 days in 2021 to 43 days in 2016 (table 2). The model predicted autumn moult occurred (between 15 September and 21 September) and ended earlier (between 2 November and 8 November) with the same moult duration of 48 days. We found some evidence of annual variation in moult initiation date with the earliest initiation in 2017 and the latest initiation in 2020 (figure 4B). However, no evidence of delayed moulting initiation was observed. When covariate influence was assessed, the model including additive effects of snow cover and mean daily temperature was better supported ($AIC_{c}wt=0.75)$ (electronic supplementary material, S1, table S3 C1). Mean daily temperature negatively influenced autumn moult initiation date; the influence of snow depth and snow cover was positive (table 1B). Autumn completion date also varied annually and there was no evidence of delayed completion in later years. Multivariate models suggested that the model with an additive effect of snow duration and maximum daily temperature was the most parsimonious $\left(AIC_{c}wt=0.89\right)$ (electronic supplementary material, S1, table S3 E1). Mean daily temperature had a strong negative influence on autumn moult completion date, whereas snow duration influenced completion date positively (table 1C).

### 3.2. Coat-colour mismatch

**Figure 5.**
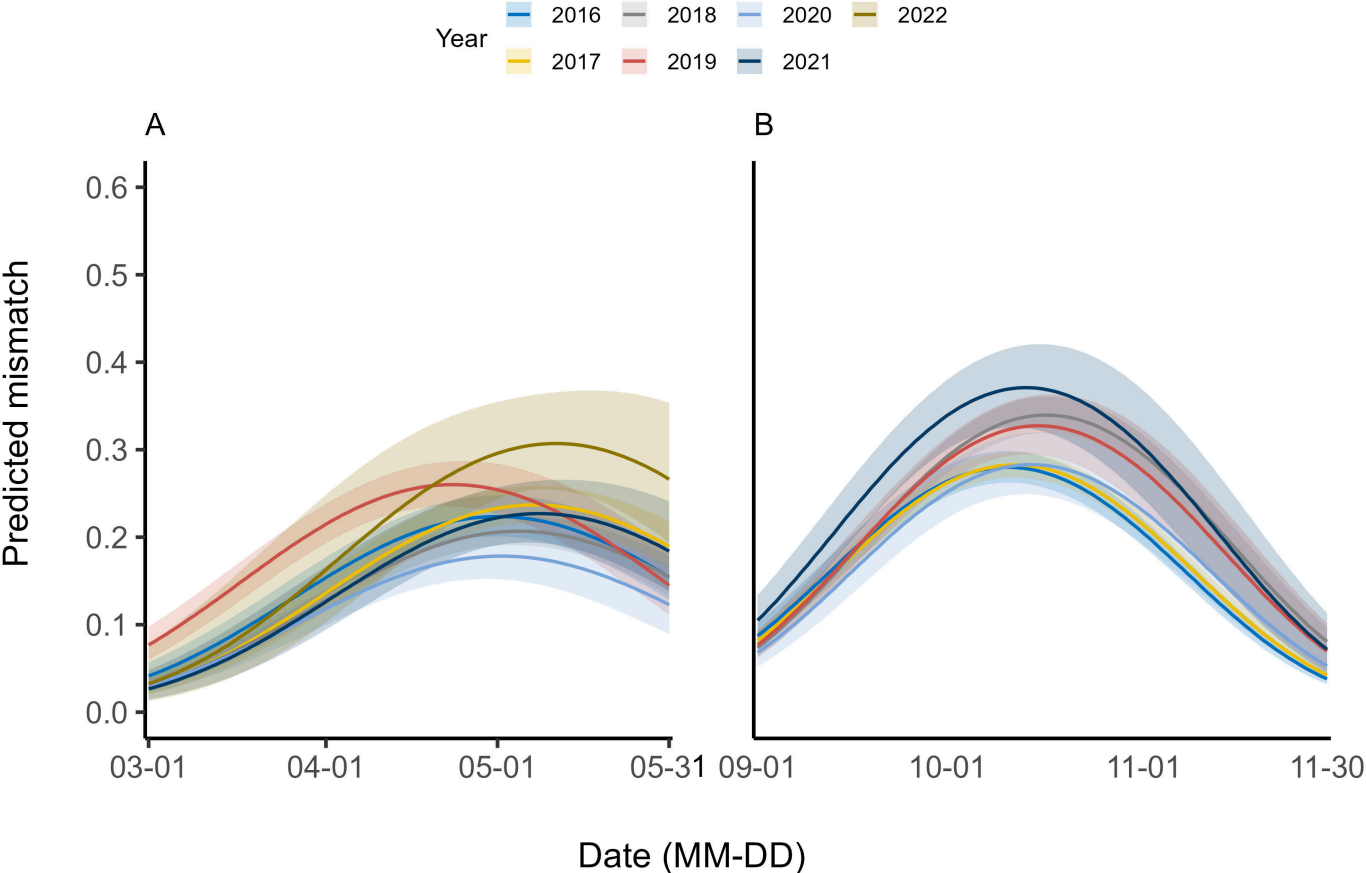
The predicted proportion of snowshoe hare coat-background colour mismatch in (A) spring and (B) autumn season as the seasonal moult progressed in Kluane lake region, Yukon, Canada, 2016–2022. Different lines represent the predicted mismatch progression for specific year and shaded region indicated 95% CIs for each year.

Spring mismatch occurs when snowshoe hares start moulting early but the background environment is predominantly white, or when the snow in the background environment melts early but hares are still white. At the beginning of the spring, all hares were white in Kluane, but mismatch increased as spring moult progressed. Generally, the peak predicted mismatch occurred around the first week of May and declined thereafter except for the year 2019 when the peak mismatch occurred around the third week of April. The earliest peak predicted mismatch occurred in 2019 (23 April) and the latest in 2022 (11 May) (figure 5A; electronic supplementary material, S2, figure S17). The lowest mismatch peak occurred in 2020 (0.18; 95% CI: 0.15–0.21), whereas the highest peak occurred in 2022 (0.31; 95% CI: 0.25–0.37). When modelling for the covariate influence, the model including the additive effects of snow cover, snow depth and mean daily temperature was the most parsimonious (electronic supplementary material, S1, table S3 H1). We found evidence of the strong quadratic influence of all covariates with a unimodal peak and strong decline in coat-colour mismatch thereafter.

Autumn mismatch occurs when coat colour and background snow are out of sync. It happens either when moulting begins before the first snowfall, or when snowfall occurs while hares are still mostly brown. In Kluane, all hares were brown at the beginning of autumn, and there was little or no mismatch between hare coat and background colour. As moulting progressed, the mismatch increased and peaked around the third week of October and progressively declined thereafter (figure 5B; electronic supplementary material, S2, figure S16). Autumn mismatch progression varied across years, with the earliest mismatch peak in 2016 (11 October) and the latest in 2018 (17 October). The lowest mismatch occurred in 2016 (0.28; 95% CI: 0.26–0.30), and the highest mismatch occurred in 2021 (0.37; 95% CI: 0.33–0.42). When we modelled the influence of multiple covariates on coat-colour mismatch, the most parsimonious model included additive effects of snow cover, snow depth and maximum daily temperature (electronic supplementary material, S1, table S3 G1). All the covariates in the top model resulted in negative coefficients indicating a concave down relationship with coat-colour mismatch (figure 5B).

Overall, coat-colour mismatch showed an increasing trend during this study, albeit with substantial variation among years and seasons (figure 2C,D). In spring, the proportion of observed high-mismatch events increased from ~8% in 2016 to ~28% in 2022 with the proportion of low-mismatch events dropping from 65% to 38% during the same period. The proportion of observed high autumn mismatch events increased from ~9% in 2016 to ~47% in 2021; the proportion of events with low mismatch declined from ~60% in 2016 to ~23% in 2021 (electronic supplementary material, S2, figure S15).

## 4. Discussion

In many high-latitude temperate and Arctic habitats, the colour of the background environment changes due to the presence of snow during winter months. At least 21 species of mammals and birds inhabiting such habitats are known to undergo seasonal moulting from brown to white during autumn, and from white to brown during spring such that camouflage is maintained as much as possible against the colour of the background environment [17,43]. Seasonal moulting ensures that the overall pelage colouration matches the natural colour of the background environment, which serves to reduce predation risk via crypsis in prey species or to improve hunting success in predators [45,46]. But the benefit of seasonal moulting depends on the initiation and completion of moulting relative to the presence of snow, which is affected by climatic factors such as ambient temperature and snow characteristics. Temperature and snow characteristics in circumpolar regions are changing due to climate change, which could influence the timing of seasonal moulting and the mismatch between the coat-colour and colour of the background environment; this can alter moult initiation and completion dates, and thus moult duration. Using 7 years of camera trapping data, we sought to quantify and discern factors affecting the timing of seasonal coat-colour moulting in an iconic boreal forest herbivore, the snowshoe hare at the core of its distributional range, the Canadian boreal forest in the Yukon.

**Figure 6.**
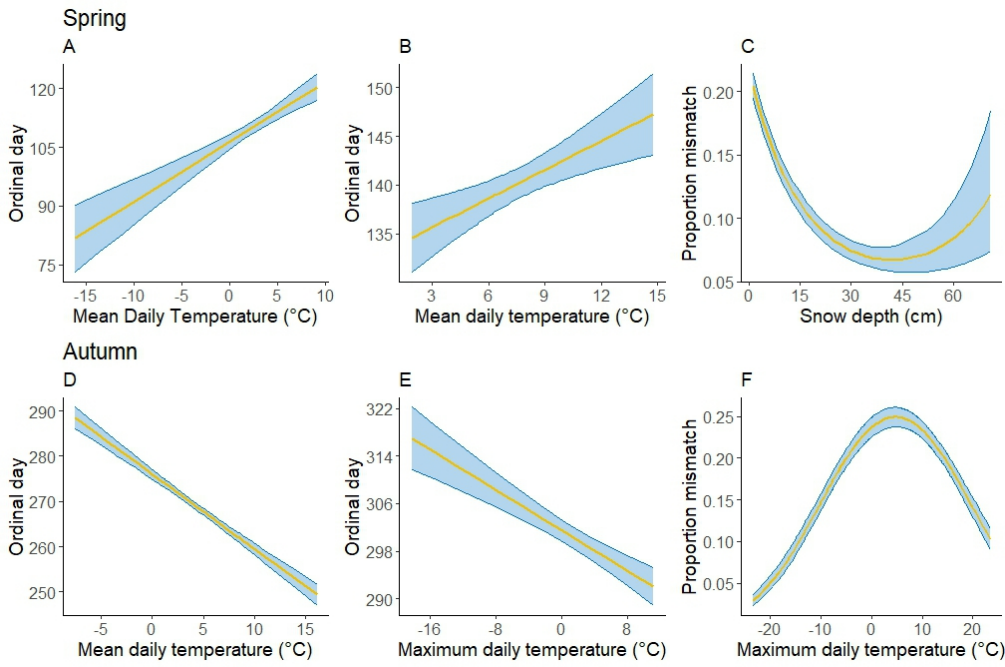
The influence of climatic covariates on spring moult initiation (A), spring moult completion (B), spring coat-colour mismatch (C), autumn moult initiation (D), autumn moult completion (E) and autumn coat-colour mismatch (F) in Kluane lake region between 2016 and 2022. The estimates used were generated from the top univariate models for each season and event (electronic supplementary material 1: table S4, A1 & B1 for autumn and spring initiation; table S5, A1 & B1 for autumn and spring completion; table S6, A1 & B1 for autumn and spring mismatch).

On average, spring moult in snowshoe hares began between 12 and 27 April and was completed between 16 and 27 May, lasting 24–38 days. Both observed and predicted moult durations varied by up to 2 weeks among years. Such interannual variation in spring moulting is not unique to snowshoe hares but has also been documented in other species, including mountain hares [47] and least weasels [48]. In 2016 and 2019, spring moult initiated earlier (~10 days) but progressed slower compared to other years (figure 3A); however, contrary to our expectation, we found no evidence of a consistent advancing trend in spring moult. This early initiation was possibly a consequence of higher mean daily temperature in those two years (electronic supplementary material, S2, figure S3). Despite this, the completion dates in those years did not differ much from other years. Contrary to our expectations and to earlier studies [21,29], snow depth was negatively associated with the initiation of spring moulting, suggesting that hares initiated moult earlier under deeper snow conditions. In contrast, snow duration exerted a positive effect, with longer snow cover delaying the onset of spring moulting. Mean daily temperature influenced both initiation and completion of moult positively suggesting delayed initiation and completion under increasing temperature, which is surprising (table 1, figure 6B,C).

Because snow cover persists longer in Kluane than at more southerly sites, spring moult begins and finishes later, and the moulting period is longer. Although spring moult began later in Kluane, completion occurred earlier than in Alberta, Montana and Colorado [21] but later than in New England and Wisconsin [21,29]. As in autumn moult, there was no consistent latitudinal pattern in the duration of the spring moult (Kluane: 30 days (this study); Alberta: 48 days [21]; New England: 51 days [21]; Colorado: 44 days [21]; Montana: 38 days [31]; Wisconsin: 38 days [29]). While higher latitude is known to lengthen moulting duration [17,44], moulting phenology studies have found mixed results on latitude-moulting duration relationship possibly highlighting the importance of local environmental conditions [21,29,47].

On average, the autumn moult started between 28 September and 3 October and completed between 5 and 11 November, with the mean moult duration ranging from 36 to 43 days. The mean initiation and completion dates varied only by 5 and 6 days, respectively, suggesting limited phenotypic plasticity in autumn moulting phenology. The predicted autumn moulting duration was longer (48 days) than the observed one and remained the same across years. There was no evidence of delayed moult initiation, earlier completion or shorter moult duration in recent years, nor was there a consistent temporal pattern in moult progression during our study period (table 2; figure 3B). Contrary to our expectations and to earlier studies [21,28], mean daily temperature and maximum daily temperature were negatively associated with autumn moult initiation and completion, respectively. In contrast, snow cover had a positive effect on moult initiation, while snow duration positively influenced moult completion (table 1; figure 5F,G). Collectively, these patterns diverge from both our predictions and previous findings [21,29].

Our study site in Kluane is near the northernmost location where seasonal moulting in snowshoe hares has been studied. In Kluane, snow arrives earlier, lasts longer and temperatures are generally lower compared to those in the southern range of snowshoe hare distribution. Thus, we expected autumn moult to start earlier, complete earlier and last shorter compared to hares inhabiting more southerly locations. Consistent with these expectations, autumn moult in Kluane on average started 2 (Wisconsin) to 27 (New England) days earlier [21,29]. Likewise, mean completion occurred 1 week to 1 month earlier in Kluane compared to Alberta, Colorado, Montana, New England and Wisconsin [21,29]. However, there was no consistent latitudinal pattern in the duration of autumn moult (Kluane: 40 days (this study); Alberta—32 days [21]; New England—46 days [21]; Colorado—42 days [21]; Montana—37 days [31]; Wisconsin—68 days [29]).

The mean coat whiteness of snowshoe hares in Kluane during autumn showed a declining trend during this study, consistent with prior observations [20,49]. Mean coat whiteness was positively influenced by snow cover in both seasons (figure 3A,B), consistent with our expectations and findings of previous studies [21,47]. However, with snow duration and snow depth both increasing in Kluane, the decrease in mean whiteness could possibly increase the coat-colour mismatch in snowshoe hares in the area.

### 4.1. Coat-colour mismatch

In spring, the hare coat-colour blended perfectly with snow until moulting began (generally between middle to late April) after which the coat-colour mismatch increased gradually until it peaked in early May; the mismatch declined progressively thereafter. While the pattern of spring mismatch was generally consistent across years, the peak predicted mismatch value and the progression of mismatch varied among years. The earliest predicted peak mismatch occurred in 2016 (23 April) and the latest in 2022 (14 May), a shift of 3 weeks, with generally latter peaks in more recent years (figure 4A). While the peak predicted mismatch in 2022 was considerably higher compared to 2016, there was no clear temporal trend. Snow depth strongly negatively influenced spring mismatch, with deeper snow resulting in reduced coat-colour mismatch consistent with our expectation. Coat-colour mismatch in spring was observed to be lowest when snow depth was ~40 cm but increased when snow depth increased further (table 1; figure 6F). Along with snow depth, the additive influence of snow cover and mean daily temperature on spring mismatch was also observed (table 1). This underscores the multifaceted nature of coat-colour mismatch and highlights the need to consider the potential influence of multiple environmental factors. Furthermore, interactions between multiple climatic variables may produce varying impacts at different spatial and temporal scales [50], suggesting a complex interplay of temperature, precipitation and other environmental factors in driving coat-colour mismatch and other phenological responses to changing climate [12,51].

Prior to the initiation of the autumn moult, all hares in Kluane were brown against brown background, so there was no mismatch. Mismatch progressively increased thereafter until it peaked in late October/early November; it declined progressively until moulting had completed and coat-colour blended with the snow-covered landscape. Whereas this pattern of autumn mismatch was generally consistent across years, the peak value and the timing of mismatch varied across years with no discernible temporal trend (figure 4B). The earliest peak predicted mismatch occurred in 2016 (11 October) and the latest in 2018 (17 October). Though the peak predicted mismatch value exhibited an increasing trend during our study, variability in observed mismatch largely overwhelmed the temporal trend (electronic supplementary material, S2 figures S16 and S17). Coat-colour mismatch in autumn was primarily influenced by the additive influence of maximum daily temperature, snow depth and snow cover. The probability of autumn coat-colour mismatch was highest when the maximum daily temperature was between 0°C and 5°C (table 1; figure 5F). The influence of both temperature and snow variables was quadratic and in the expected direction [17].

Our findings on increasing mismatch generally align with many of the results of other similar studies across the southern part of the hare’s distributional range [21] but some notable divergences do occur. Generally, the increased mismatch is attributed to the decreasing snow duration and snow cover [21,23,29]; in Kluane, however, mismatch increased despite increasing snow duration and depth. This is likely related to the decrease in mean coat whiteness in snowshoe hare over past decades ([20] and this study), possibly due to declining snow duration and depth between 2005 and 2016 [52]. However, the decreased mean coat whiteness, under the current snow regime, causes hares to remain mismatched.

Coat-colour mismatch represents a critical challenge for snowshoe hares in a warming climate, as its implications for survival are profound [53]. Evidence indicates reduction of weekly survival rates for mismatched hares by up to 12% [19,20,53]. While some species exhibit behavioural plasticity in response to predation risk, as seen in red king crabs *Paralithodes camtschaticus* in Alaska and guppies *Poecilia reticulata* in Trinidad [54–56], there is limited evidence of predation-risk-induced phenotypic or behavioural plasticity in snowshoe hares, especially in its ability to use habitat features for camouflage [30,31,57]. This is critical given that by altering habitat use patterns, hares could potentially reduce the predation risk. For example, predation risk may be lower in larger and wooded habitat patches compared to open and smaller habitat patches [19]; and effective use of shrub or rock cover can also aid in predator avoidance [58,59]. While hares are known to change their diel activity to reduce predation due to mismatch [57], there is as yet little evidence for behavioural responses in snowshoe hares that aid in better camouflage against predators [21,30,31,60]. While some evidence of coat-colour mismatch providing thermoregulation benefits to snowshoe hare have been found [49], the primary objective of coat-colour is to enhance crypsis for better survival [17,45]. This renders the mismatched snowshoe hares more vulnerable to predation compared to camouflaged ones. In an ecosystem where many species of mammalian and avian predators depend on snowshoe hare abundance for reproductive success, an increased mismatch can potentially dampen snowshoe hare population cycles, which could have a cascading effect on boreal ecosystem [61,62].

## 5. Conclusions

Our 7-year study conducted at the highest latitude to date in the distributional range of snowshoe hares revealed that the dates for autumn moult initiation as well as completion varied little across years (about a week), and average moult duration ranged from 36 to 43 days. Spring moult initiation and completion dates were more variable, with moult duration lasting from 24 to 38 days. Consistent with our expectations: (i) the average autumn coat-colour whiteness exhibited a declining trend, and coat-colour mismatch showed an increasing trend; (ii) autumn moult in Kluane on average started and completed earlier; and (iii) spring moult in Kluane started earlier than that in southern part of the range, but there was no evidence of a discernible latitudinal gradient in moult duration. Contrary to our expectations, there was no evidence of delayed autumn moult initiation or completion, or advanced spring moult phenology. Also, the influence of effects of climatic covariates on aspects of seasonal moulting phenology was not in the expected direction. For example, increasing mean daily temperature advanced initiation of autumn moulting contrary to our expectation that it should delay. The influence of snow depth and mean daily temperature on spring initiation and completion was also the opposite of predicted direction.

We offer three possible explanations for some of the unexpected results from our study. First, our study lasted only 7 years, with limited variation in temperature and snow conditions, possibly insufficient to meaningfully impact seasonal moulting phenology. Second, we relied on climatic data collected from the Haines Junction weather station, Yukon, which may not have accurately represented microclimatic conditions in snowshoe hare habitats. Evidence suggests local microclimates could play a greater role in moulting phenology than regional climatic patterns [17]. Finally, moulting phenology may be influenced by interactive effects of many intrinsic and extrinsic environmental factors. We considered only a handful of potentially important variables for which data were available. Longer-term studies utilizing intrinsic (e.g. genetic) and high-resolution habitat and microclimatic data would be needed to discern how a multitude of factors might interact to influence seasonal moulting phenology in snowshoe hares.

Overall, our results and those of earlier studies indicate coat-colour mismatch is increasing, and this can have profound impact on snowshoe hare survival and population dynamics [19–21]. Because high winter mortality is the primary driver of hare population crashes [63], increased mismatch can dampen snowshoe hare population cycles, which can adversely affect reproductive success of a suite of mammalian and avian predators. While, phenotypic or behavioural plasticity may help mitigate some of the negative effects of mismatch, detailed investigations into the phenotypic and behavioural plasticity of snowshoe hares’ response to increasing mismatch, and its effect on the interaction between snowshoe hares (or other seasonal moulting prey species) and their main predators would be essential to fully understand how changing climate and increasing mismatch affect demography and population dynamics of seasonally moulting birds and mammals.

## Data Availability

The datasets supporting this article have been uploaded as part of the electronic supplementary material at Zenodo repository [64]. Supplementary material is available online [65].
